# Author Correction: Pentavalent and tetravalent uranium formation via glycerol-stimulated bacteria in mine water

**DOI:** 10.1038/s41467-026-74738-x

**Published:** 2026-06-22

**Authors:** Antonio M. Newman-Portela, Kristina O. Kvashnina, Elena F. Bazarkina, André Rossberg, Frank Bok, Sean Ting-Shyang Wei, Andrea Kassahun, Thorsten Stumpf, Johannes Raff, Mohamed L. Merroun, Evelyn Krawczyk-Bärsch

**Affiliations:** 1 Institute of Resource Ecology, Helmholtz-Zentrum Dresden-Rossendorf, Dresden, Germany; 2 Faculty of Science, Department of Microbiology, University of Granada, Granada, Spain; 3 The Rossendorf Beamline (BM20-ROBL), European Synchrotron Radiation Facility, Grenoble, France; 4 WISMUT GmbH, Chemnitz, Germany; 5 Present Address: Institut des Sciences de la Terre (ISTerre), Grenoble, France

**Keywords:** Environmental chemistry, Pollution remediation

Correction to: *Nature Communications* 10.1038/s41467-026-72560-z, published online 4 May 2026

In the version of this article initially published, there were typographical errors in units discussed in eight instances, where the current values listed for “mg L^–1^” appeared originally as “mg mL^–1^.” Text has been corrected in the first and second paragraphs of the Introduction, first paragraph of the “Geochemistry and kinetics of U reduction in microcosms” subsection, tenth paragraph of the “Spectroscopic and microscopic analysis of bioreduced U(VI)” subsection, first paragraph of the “Implications for U immobilisation and remediation” subsection and first paragraph of the “Metatranscriptomic analysis of Schlema-Alberoda mine water” subsection. The text has been updated in the HTML and PDF versions of the article.

