# Supplementary material for: Pentavalent and tetravalent uranium formation via glycerol-stimulated bacteria in mine water
Source: Nat Commun. 2026 May 4;17:4030. doi: 10.1038/s41467-026-72560-z (PMC13139381; doi:10.1038/s41467-026-72560-z)
Supplement: Supplementary file 1 — Supplementary Information [file 41467_2026_72560_MOESM1_ESM.pdf]

Supplementary Information

Pentavalent and Tetravalent Uranium Formation via Glycerol-Stimulated Bacteria in Mine Water

Antonio M. Newman-Portela [1,2,*], Kristina O. Kvashnina [1,3,*], Elena F. Bazarkina [1,3,†], André Rossberg [1,3], Frank Bok [1], Sean Ting-Shyang Wei [1], Andrea Kassahun [4], Thorsten Stumpf [1], Johannes Raff [1], Mohamed L. Merroun [2,*], Evelyn Krawczyk-Bärsch [1,*]

[1] Helmholtz-Zentrum Dresden-Rossendorf, Institute of Resource Ecology, Dresden, Germany

[2] Department of Microbiology, Faculty of Science, University of Granada, Granada, Spain

[3] The Rossendorf Beamline (BM20-ROBL), European Synchrotron Radiation Facility, Grenoble, France

[4] WISMUT GmbH, Chemnitz, Germany

† Present address: Institut des Sciences de la Terre (ISTerre), Grenoble, France

* Author for correspondence: a.newman-portela@hzdr.de / kristina.kvashnina@esrf.fr / merroun@ugr.es / e.krawczyk-baersch@hzdr.de

Supplementary Methods 1. Oxidation State Quantification via Iterative Target Factor Analysis (ITFA)

The quantification of U(IV), U(V) and U(VI) fractions is based on iterative target factor analysis (ITFA). The details are explained in Rossberg et al.[45]. Briefly, ITFA is a multivariate analysis technique commonly used in spectroscopy in order to decompose complex spectral datasets into meaningful chemical components by resolving overlapping signals.

In the first step, eigenanalysis, a mathematical technique used in linear algebra to study linear transformations, is applied to determine the rank, i.e., the number of linearly independent spectral components. The number of independent factors is identified using the eigenvalues and a semi-empirical indicator function. The number of primary factors is determined when the indicator function reaches its minimum value. This is principal component analysis (PCA).

In the second step, iterative target testing (ITT) is applied to the spectra of samples and reference compounds of pure U(IV), (V) and (VI) and the number of pure components is fixed to three. The ITT procedure uses concentration test vectors for non-orthogonal rotation and determines factor concentrations independently. The final model is a linear combination fit (LCF) of independent components spectra found by ITT.

Final models are compared to the experimental data in the Figure S13.

The errors identified by ITFA analysis are difficult to estimate due to uncertainty and the potential presence of multiple forms of each oxidation state in such materials. Based on the common E positions of U(IV) compounds, the error for U(IV) is estimated to be around 5%. The limited number of spectra available for U(V) compounds, the close E positions of U(V) and U(VI), and the known strong variations in the spectra of different U(VI) compounds contribute to an expected uncertainty of approximately 10% for the quantification of U(V) and U(VI).

However, the assignment of U(V) was validated against $UMoO_5$ and further validated by comparison with $NaUO_3$, KUO_3 and U_4O_9[71–75]. These well-characterised U(V) compounds exhibit nearly identical HERFD-XANES peak positions and envelope shapes at the M_4-edge, thereby providing strong confidence in the spectral identification of pentavalent uranium in this study.

In our case, ITFA-derived fractions occasionally deviated slightly from 100% (typically 103–107%), which falls within the estimated uncertainty. For clarity, values were renormalised to sum exactly to 100%.

Supplementary Methods 2. Analytical Methods and Physicochemical Measurements

Mine water samples (50 L) were collected in June 2022 at the inlet of the treatment plant in the Wismut GmbH Schlema-Alberoda mine. The sampling pipes were purged to prevent contamination and preserve the water's chemical and microbiological integrity. *In situ* measurements of pH and redox potential were taken. The sample was then transported at 4 °C for immediate use for U reduction microcosm experiments.

Physicochemical parameters, including pH, E_h, and the concentrations of U, Fe, As, and SO_4^{2-}, were monitored weekly throughout the microcosm experiment. pH was measured using a WTW 3110 metre with a BlueLine 16 microelectrode (Schott Instruments, Germany). E_h values were determined with a micro redox electrode featuring a platinum ring (Mettler-Toledo InLab, Spain). Total dissolved cations and anions were analysed at the beginning (day 1) and end (day 130) of the experiment using inductively coupled plasma mass spectrometry (ICP-MS, ELAN 9000, PerkinElmer, Germany) and high-performance ion chromatography (HPIC, Dionex Integrion, Thermo Fisher Scientific, USA), respectively. Samples for ICP-MS were acidified with nitric acid (HNO_3) prior to analysis. Total inorganic/organic carbon (TIC/TOC), dissolved organic carbon (DOC), and total nitrogen were quantified using a Multi N/C 2100S (Analytik Jena, Germany).

The analytical data from the biostimulation experiment were used to calculate the predominance fields of possible U species. A Pourbaix diagram was generated utilising the geochemical speciation code Geochemist's Workbench, version 18.0.3[38]. The PSI Chemical Thermodynamic Database 2020[39] served as the thermodynamic database for this calculation.

Supplementary Methods 3. Determination of nanoparticle dimensions and Equivalent Circular Diameter (ECD)

Size analysis was performed on HRTEM micrographs using ImageJ (version 1.54g; NIH). Given that the NPs exhibited variable morphologies, the equivalent circular diameter (ECD) was used as a consistent metric to compare particles sizes independently of shape[81]. NPs outlines were traced, and the following parameters were recorded: long axis (maximum Feret diameter, nm), short axis (minimum Feret diameter, nm), and projected area (A, nm^2). From the projected area, the ECD (nm) was calculated as:

$$ECD = \sqrt{\frac{4A}{\pi}}$$

Histograms of ECD were constructed in R (version 4.5.1) using custom scripts. Both frequency- and density-based histograms were generated, and a log-normal distribution was fitted to capture the skewed nature of the size distribution. Descriptive statistics (mean, standard deviation, median, interquartile range, minimum, and maximum) were also computed. The complete dataset (n = 160 particles) is available in Supplementary Table S4 and the histogram in Figure S9. A second histogram (Fig. S10), showing the distribution of nanoparticles by mineral phase and Miller index (hkℓ), was also constructed in R (version 4.5.1). This analysis included n = 231 nanoparticles.

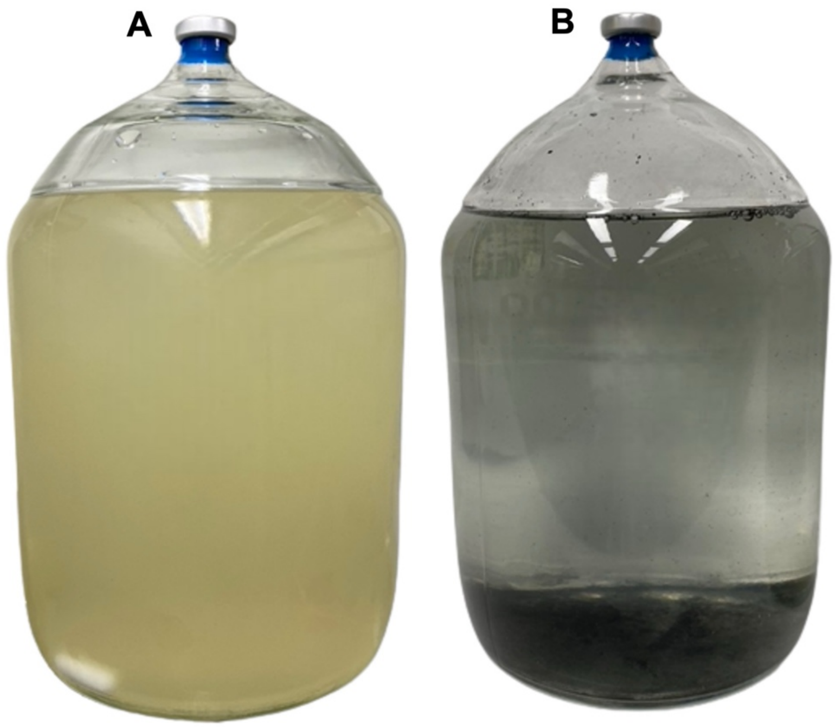

Supplementary Fig. 1. Colour transition in the glycerol-amended microcosms during the U(VI) reduction experiment. (A) At the beginning of the incubation (day 1), the mine water appeared light yellow. (B) After 130 days, a black precipitate formed at the bottom of the bottles, while the overlying water remained clear.

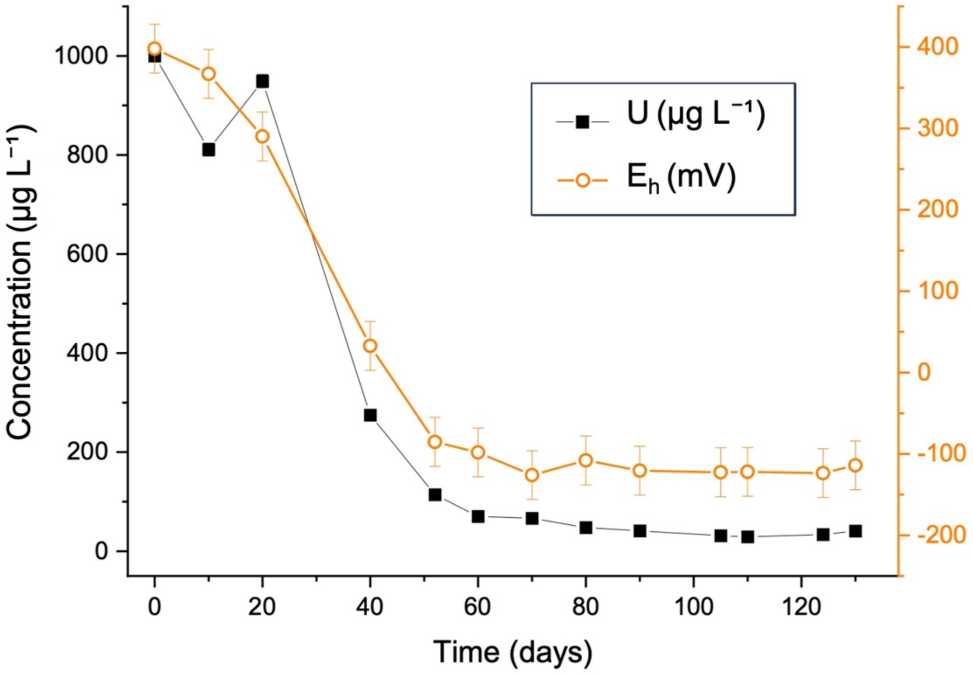

Supplementary Fig. 2. Evolution of U(VI) concentration and redox potential. Monitoring of U(VI) concentration (μg L^{-1}) and redox potential (E_h, mV) in glycerol-amended microcosms (10 mM) over 130 days. The error bars indicate one standard deviation (n = 3). However, the error bars are too small to be visible in the plot.

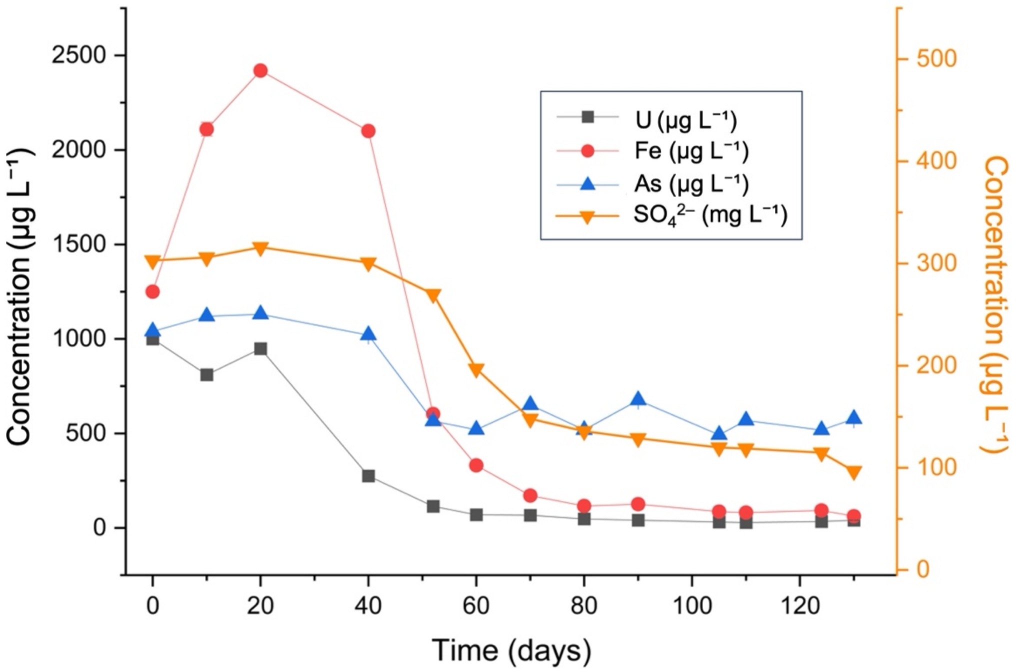

Supplementary Fig. 3. Evolution of Fe, As, U(VI) and sulphate concentrations. Monitoring of Fe, As, and U(VI) concentrations (μg L^{-1}) and SO_4^{2-} (mg L^{-1}) in microcosm experiments amended with 10 mM glycerol for 130 days. The error bars correspond to one standard deviation (n = 3). The error bars are too small to be visible in the figure.

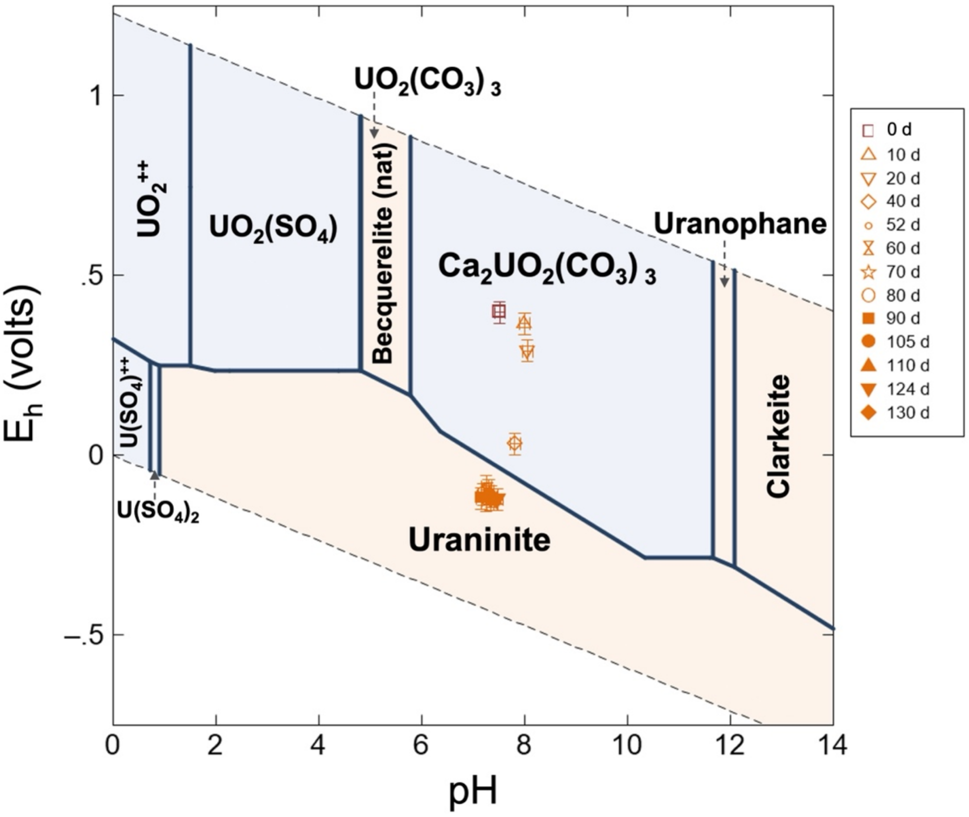

Supplementary Fig. 4. Pourbaix diagram of the Schlema-Alberoda mine water microcosms. Diagram generated using thermodynamic calculations with the Geochemist's Workbench geochemical speciation code (module Act2, version 18.0.3), the PSI Chemical Thermodynamic Database 2020, and corresponding analytical data.

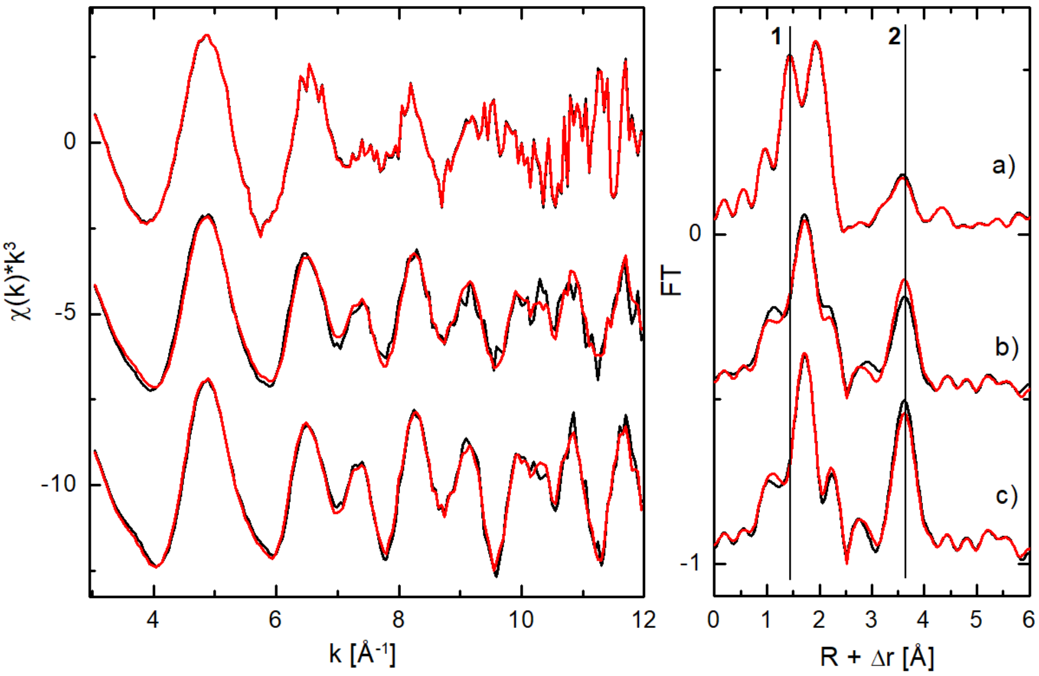

Supplementary Fig. 5. EXAFS spectra of uranium species during microcosm incubation. U L_3-edge k^3-weighted EXAFS spectra (left) and corresponding Fourier-transforms (FT, right) from the black precipitates of the Schlema-Alberoda mine water microcosms (10 mM glycerol, anaerobic conditions), collected at defined sampling points corresponding to conditions at which the dissolved U(VI) concentration in the aqueous supernatant had decreased by approximately 20% (a), 60% (b) and 90% (c) relative to the initial value. These sampling points were reached after ~10, ~30 and ~55 days of incubation, respectively. Experimental spectra are shown in black and ITFA reproductions in red.

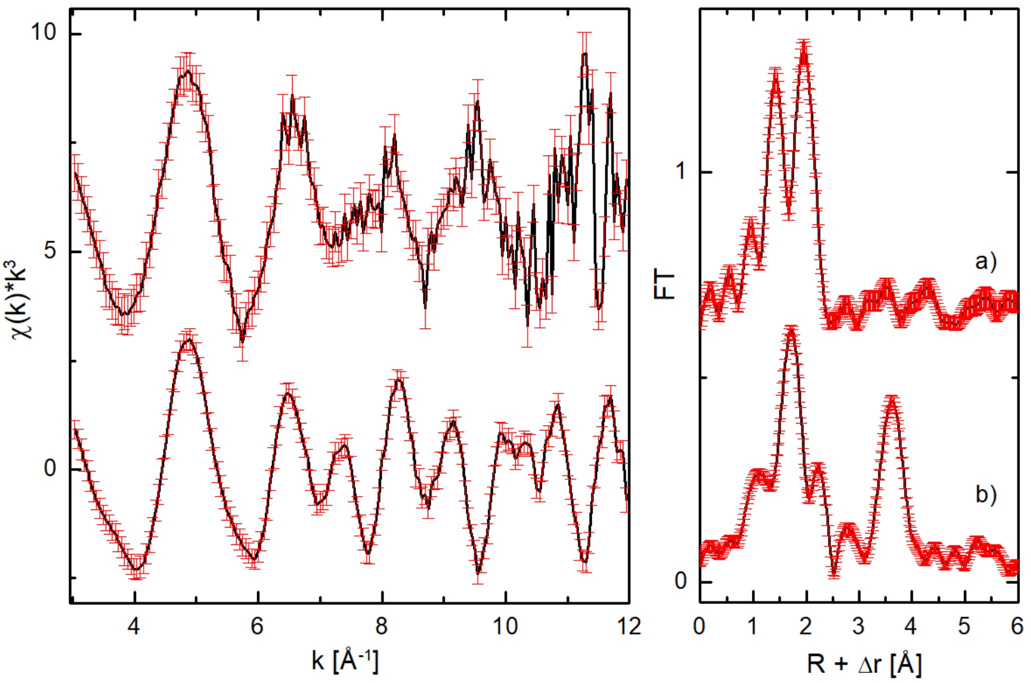

Supplementary Fig. 6. ITFA-isolated uranium species identified by EXAFS. U L_3-edge k^3-weighted EXAFS spectra (left) and corresponding Fourier-transforms (FT, right) of the ITFA isolated U species (black) with estimated standard deviations (red). U(V/VI) species a), uraninite-like phase b).

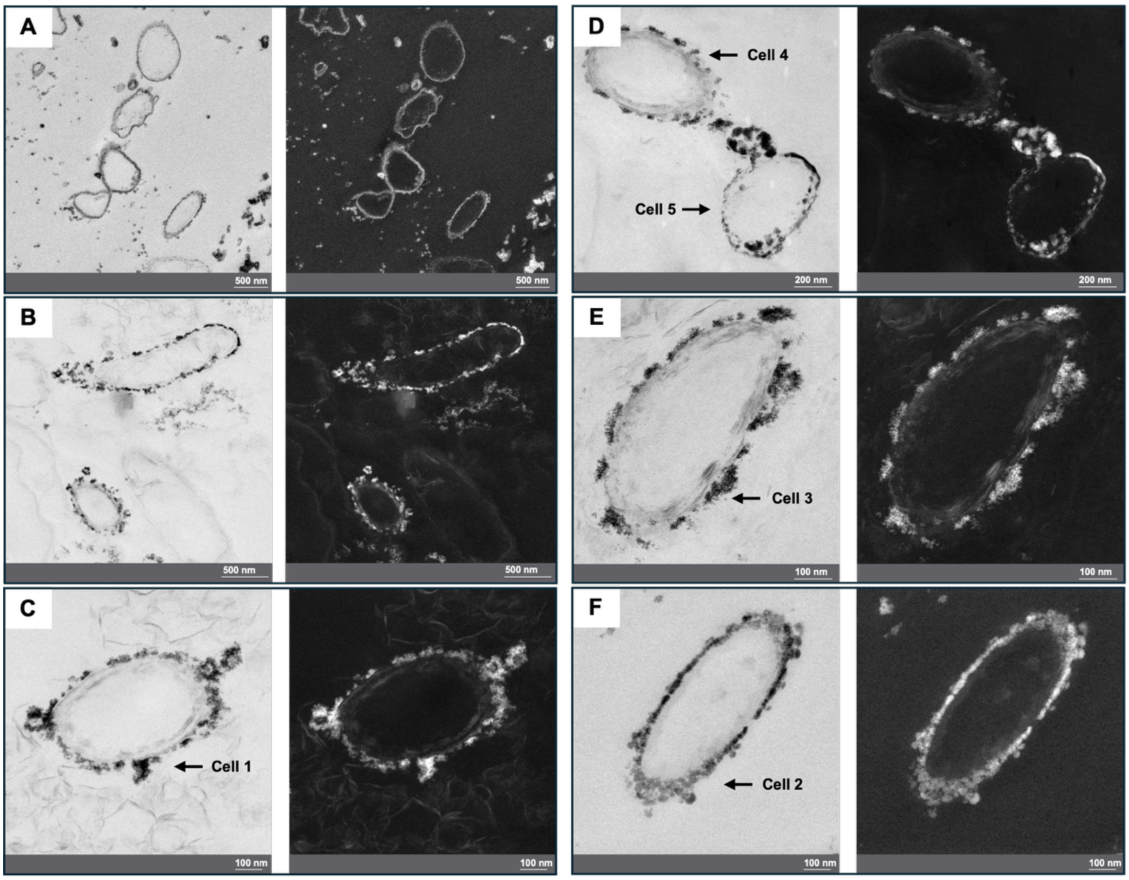

Supplementary Fig. 7. TEM and STEM images showing U nanoparticle aggregates. Panoramic bright-field transmission electron microscopy (BF-TEM) and high-angle annular dark-field scanning transmission electron microscopy (HAADF-STEM) images showing consistent U nanoparticles agglomerates formation across multiple cells. Images A–B: Panoramic BF-TEM (left) and HAADF-STEM (right) images showing complete cells within the same field of view. In HAADF-STEM, the agglomerates containing U nanoparticles appear as bright features that show a consistent cellular distribution across independent cells. Images D–F: High-magnification images of five representative cells from the set used for quantitative analysis (Cells 1–5), showing the morphology and localisation of the electron-dense agglomerates along the cell.

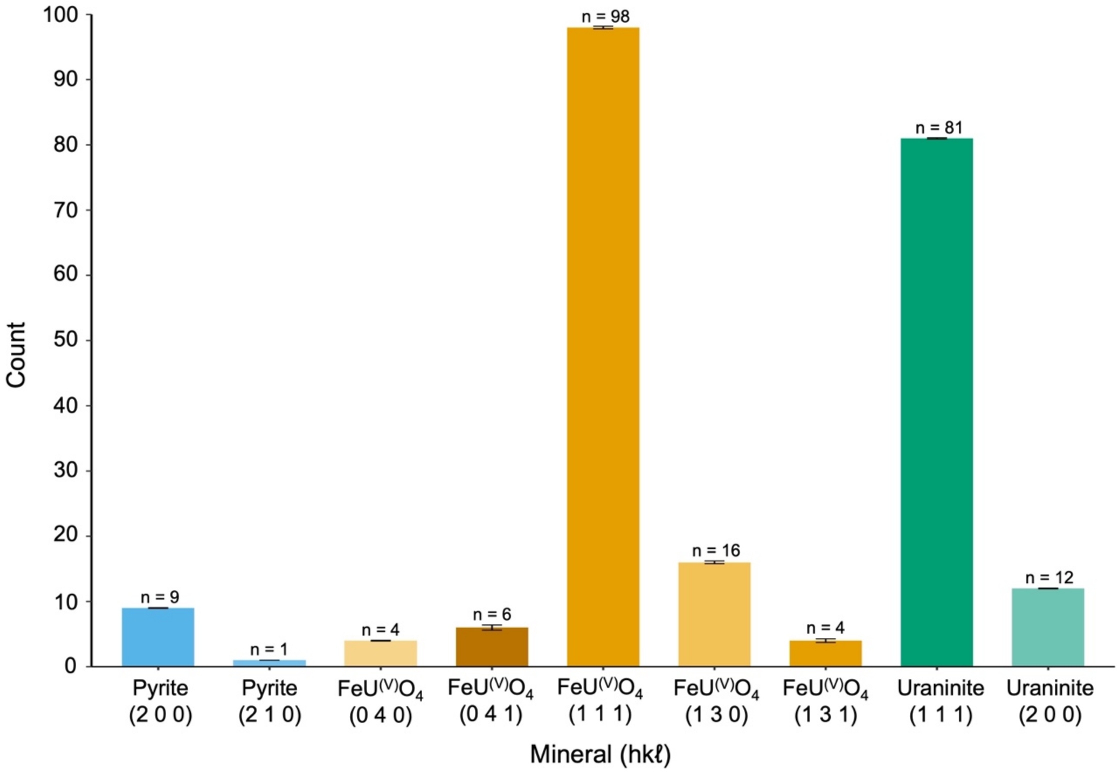

Supplementary Fig. 8. Distribution of nanoparticles by mineral type and crystallographic plane. Histogram of the number of nanoparticles (NPs) identified by mineral and Miller index (hkℓ). Each bar shows the count (n) of NPs in which that (hkℓ) was recognized, the n is printed above each bar. Thin vertical lines indicate the standard deviation (SD) of the d-spacing (Å). Error-bar length is proportional to SD and has been linearly scaled to the count axis. Base colours encode the mineral (Pyrite, $FeU^{(V)}O_4$, Uraninite) and shades distinguish (hkℓ).

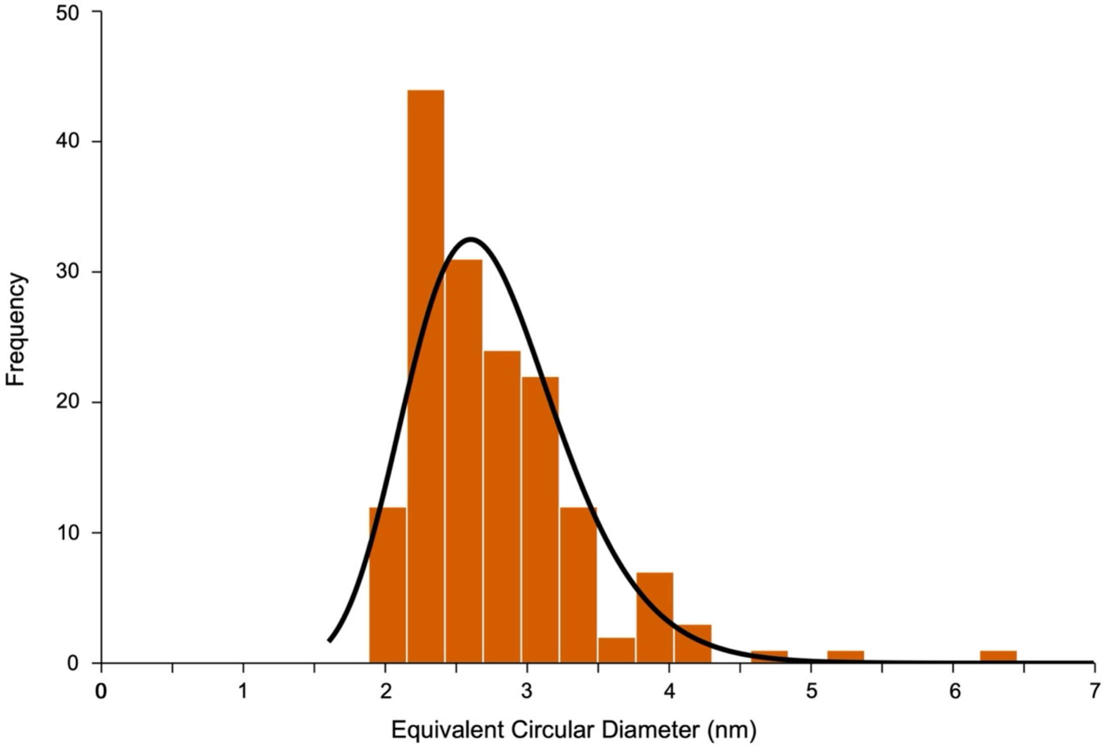

Supplementary Fig. 9. Size distribution of U nanoparticles. Histogram of Equivalent Circular Diameter (ECD) distribution of the U nanoparticles obtained from TEM measurements (n = 160). Each bar represents the number of particles, while the black line corresponds to the fitted log-normal distribution. Statistical parameters of the distribution were: mean = 2.77 nm, standard deviation = 0.63 nm, median = 2.62 nm, interquartile range = 0.73 nm, minimum = 2.00 nm, and maximum = 6.44 nm.

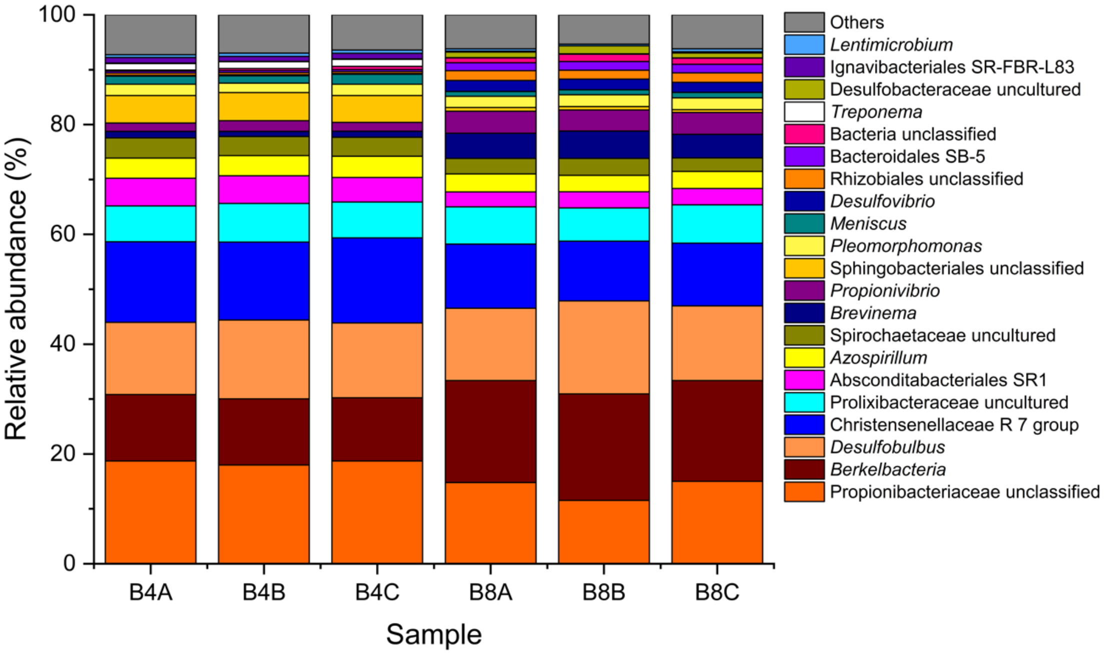

Supplementary Fig. 10. Taxonomic distribution of microbial communities. Barplot of the taxonomic distribution of bacterial diversity in the glycerol-amended microcosms B4 and B8. These microcosms exhibited similar geochemical conditions and were sampled at the end of the 130-day incubation, when more than 90% of U(VI) was removed from the supernatant. Each microcosm was analysed with three replicates (n = 3). Only genera detected in all replicates with >0.5% relative abundance were included. The remaining genera were grouped as “others”.

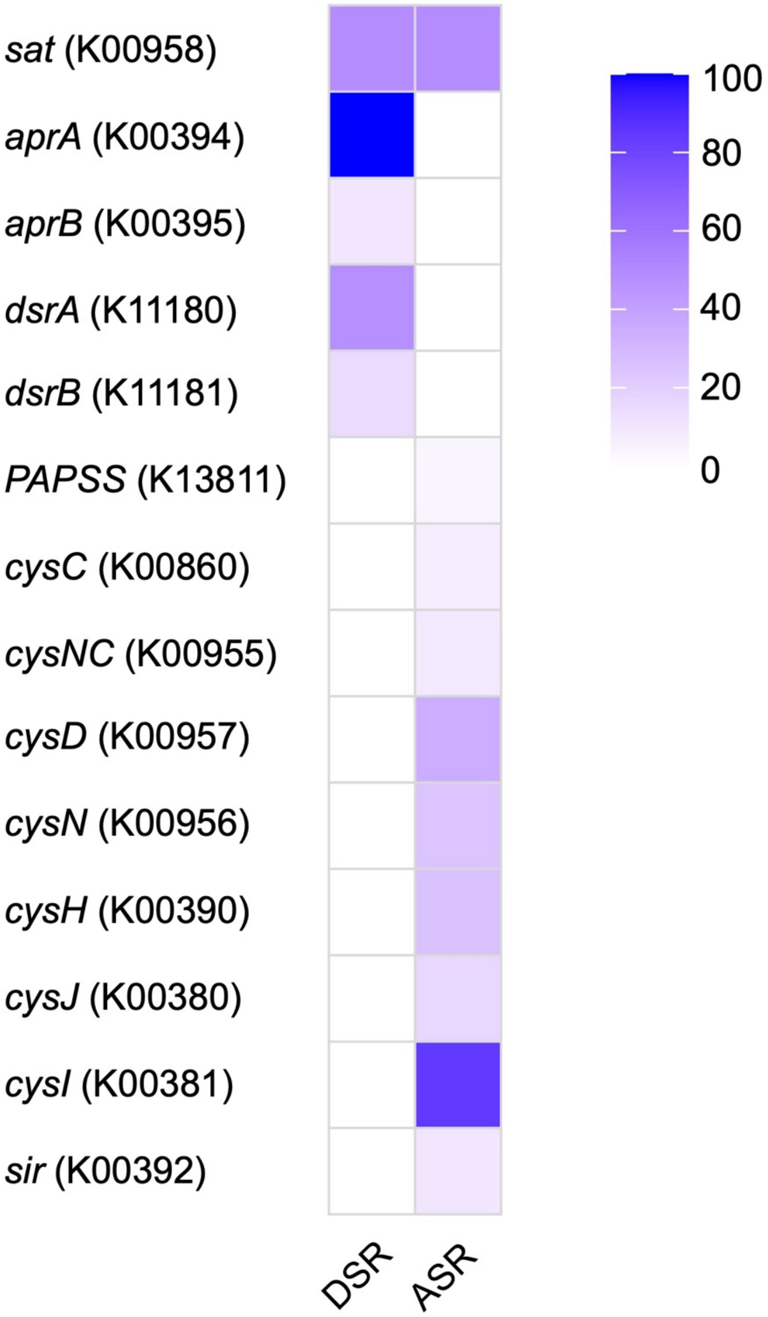

Supplementary Fig. 11. Abundance of genes involved in sulphate reduction. Heatmap of abundance of genes involved in dissimilatory (DSR) and assimilatory (ASR) sulphate reduction. Each row corresponds to a functional gene (KEGG orthology in brackets). Colour intensity denotes transcript counts ranging from 0 (white) to 100 (dark blue). The number of transcripts is listed in Table S6.

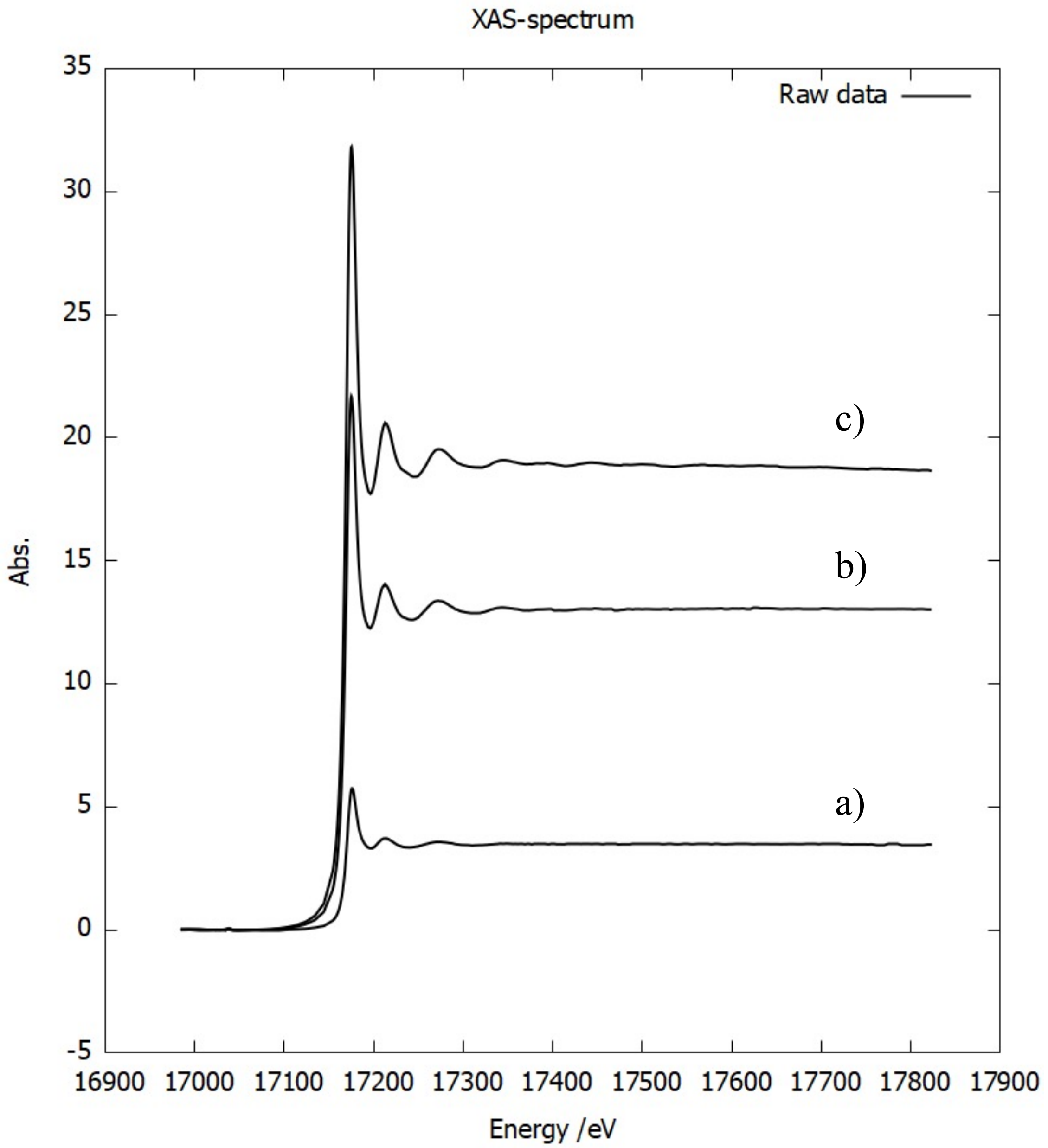

Supplementary Fig. 12. Raw U L_3-edge XAS spectra during U reduction. Raw U L_3-edge XAS spectra from black precipitates collected from the Schlema-Alberoda mine water microcosms (10 mM glycerol, anaerobic conditions) at defined sampling points corresponding to approximately ~20%, ~60% and ~90% decreases in dissolved U concentration in the aqueous supernatant, reached after ~10 days (a), ~30 days (b) and ~55 days (c) of incubation, respectively.

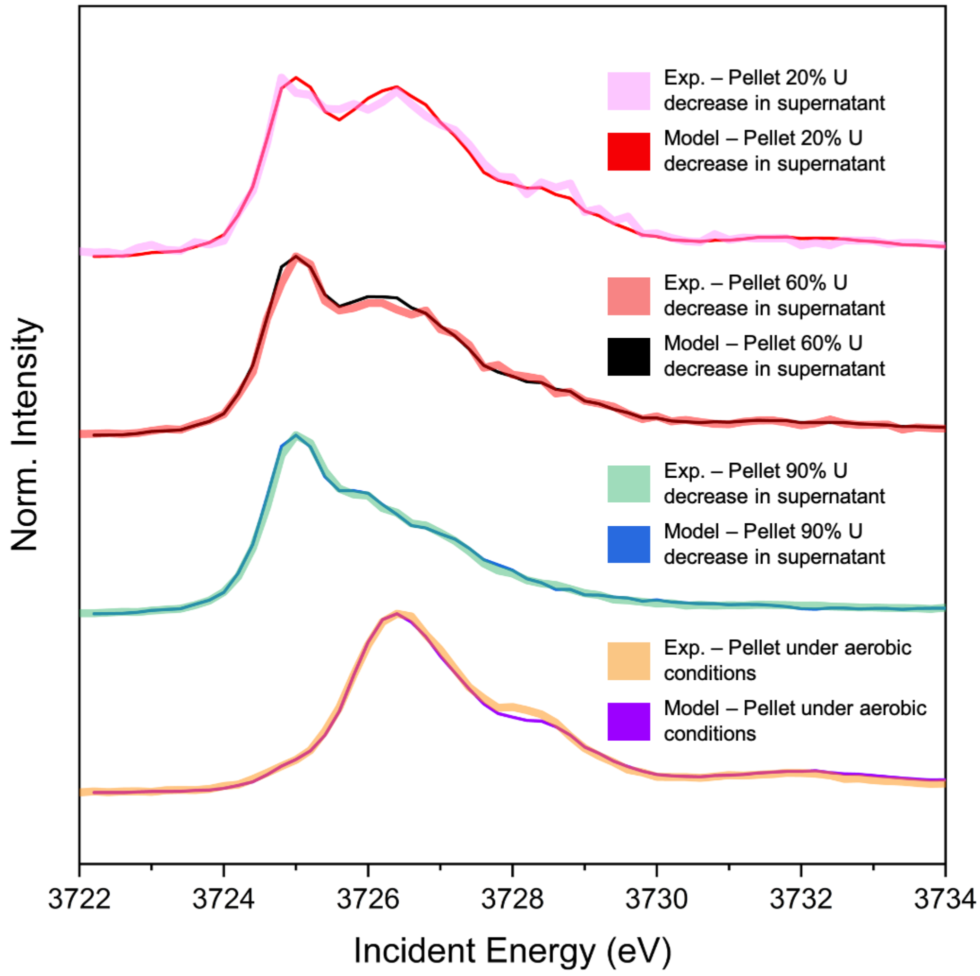

Supplementary Fig. 13. HERFD-XANES spectra and ITFA fits of uranium species. U M_4-edge HERFD-XANES spectra and corresponding ITFA model fits of black precipitates collected at defined sampling points corresponding to conditions at which the dissolved U(VI) concentration in the aqueous supernatant had decreased by approximately 20%, 60% and 90%, reached after ~10, ~30 and ~55 days of incubation, respectively (see Fig. S2), together with a sample subsequently exposed to aerobic conditions for four weeks. ITFA models were obtained by linear combination fitting using reference spectra with the indicated proportions.

Supplementary Table 1. Chemistry of the mine water during the monitoring of the U(VI)-reduction experiment.

Time (days)	0	10	20	40	52	60	70	80	90	105	110	124	130
E_h [mV]	398	367	290.9	32.7	–85.2	–98.1	–126	–108	–120.6	–122.5	–122	–123.6	–114
pH	7.5	8.0	8.1	7.8	7.3	7.3	7.3	7.2	7.2	7.3	7.4	7.5	7.4
Cations [mg L^{-1}]													
Fe	3.21	2.11	2.42	2.10	0.60	0.33	0.17	0.12	0.13	0.09	0.08	0.09	0.06
As	1.3	1.12	1.13	1.02	0.57	0.52	0.65	0.52	0.68	0.49	0.57	0.52	0.58
U	1.00	0.81	0.95	0.28	0.11	0.07	0.07	0.05	0.04	0.03	0.03	0.03	0.04
Anions [mg L^{-1}]													
SO_4^{2-}	303	306	316	301	270	197	148	136	129	120	119	115	96.8

E_h: Redox potential.

Supplementary Table 2. Physico-chemical characterisation of Schlema-Alberoda mine water in July 2022 (SA) and at the end of the microcosm experiments after 130 days (SA+G, SAC, ASA+G).

	SA	SA+G	SAC	ASA+G
pH	7.5 ±0.1	7.4 ±0.1	7.4 ±0.1	7.7 ±0.1
E_h [mV]	398 ±0.1	–114 ±0.1	68 ±30	135 ±30
Temp. (ºC)	25.5 ±1	28 ±1	28 ±1	28 ±1
Cations [mg L^{-1}]				
Na	89.3 ±0.2	90.03 ±1.57	84.3 ±0.5	85.66 ±1.39
Mg	54.2 ±0.5	51.13 ±1.57	45.3 ±1	47.87 ±0.76
Al	< 0.001	< 0.001	< 0.01	< 0.01
Si	7.5 ±0.1	9.01 ±0.26	8.3 ±0.1	8.94 ±0.16
P	< 0.01	< 0.1	< 0.1	< 0.1
K	9.3 ±0.05	10.22 ±0.02	10.1 ±0.16	9.97 ±0.22
Ca	122 ±0.6	81.53 ±2.1	58.9 ±1.1	48.93 ±1.03
Mn	1.4 ±0.05	1.19 ±0.002	0.12 ±0	0.12 ±0.002
Fe	3.21 ±0.02 *	0.17 ±0.1	2.2 ±0.1	0.33 ±0.02
As	1.3 ±0.02 *	0.97 ±0.04	1.4 ±0.005	0.78 ±0.04
Ba	0.03	0.03	0.03	0.02
Th	< 0.001	< 0.001	< 0.001	< 0.001
U	1.0 ±0.01	0.05 ±0.01	0.75 ±0.005	0.64 ±0.02
Anions [mg L^{-1}]				
Cl^-	51.6 ±0.25	< 0.05	50.3 ±0.1	48.1 ±0.1
NO_2^-	< 0.5	< 0.05	< 0.05	< 0.05
NO_3^-	0.1 ±0.01	< 0.05	< 0.05	< 0.05
PO_4^{3-}	< 0.5	< 0.05	< 0.05	< 0.05
SO_4^{2-}	302.0 ±1.0	94.6 ±1.0	289.5 ±3.5	291.7 ±1.0
[mg L^{-1}]				
TIC	56.8 ± 1.1	75.6 ±1.3	68.4 ±0.4	64.9 ±0.5
TOC	1.9 ±0.1	280.2 ±0.8	3.1 ±0.27	451.4 ±1.1
DOC	1.8 ±0,17	285.3 ±0.8	9.33 ±1.07	463.1 ±0.9
TN	< 0.1	< 0.5	< 0.5	< 0.5

SA: Schlema-Alberoda mine water during sampling; SA+G: Schlema-Alberoda mine water + 10 mM glycerol; SAC: Schlema-Alberoda mine water after 130 days; ASA+G: sterilized (autoclaved) Schlema-Alberoda mine water + 10 mM glycerol; E_h: Redox potential; Temp: Temperature; TIC: total inorganic carbon. TOC: total organic carbon, TN: total nitrogen; standard deviation with n = 3; * analysed by Wismut GmbH (ICP analysis without centrifugation prior to acidification).

Supplementary Table 3. Expression of transcripts in Schlema-Alberoda mine water via metatranscriptome involved in dissimilatory sulphate reduction (DSR) and assimilatory sulphate reduction (ASR) based on KEGG.

KEGG Pathway	Gene (KO)	Protein	No. of transcripts
**Dissimilatory sulphate reduction (DSR)**	*sat* (K00958)	Sulphate adenylyltransferase	49
	aprA (K00394)	APS reductase, subunit A	100
	aprB (K00395)	APS reductase, subunit B	10
	dsrA (K11180)	Dissimilatory sulphite reductase, subunit A	48
	dsrB (K11181)	Dissimilatory sulphite reductase, subunit B	15
**Assimilatory sulphate reduction (ASR)**	*PAPSS* (K13811)	PAPS synthase	4
	sat (K00958)	Sulphate adenylyltransferase	49
	cysC (K00860)	APS kinase	7
	cysNC (K00955)	Sulphate adenylyltransferase subunit	9
	cysD (K00957)	Sulphate adenylyltransferase subunit	35
	cysN (K00956)	Sulphate adenylyltransferase subunit	25
	cysH (K00390)	PAPS reductase	26
	cysJ (K00380)	Sulphite reductase (flavoprotein)	17
	cysI (K00381)	Sulphite reductase (hemoprotein)	85
	sir (K00392)	Assimilatory sulphite reductase	10
