# Supplementary material for: Pentavalent and tetravalent uranium formation via glycerol-stimulated bacteria in mine water
Source: Nat Commun. 2026 May 4;17:4030. doi: 10.1038/s41467-026-72560-z (PMC13139381; doi:10.1038/s41467-026-72560-z)
Supplement: Supplementary file 2 — Description of Additional Supplementary File [file 41467_2026_72560_MOESM2_ESM.pdf]

Description of Additional Supplementary Files

Supplementary Data 1. Per-cell assignment of nanoparticles identified by HRTEM: Sample, Miller index (hkℓ), mineral phase, and interplanar d-spacing (d (Å)). Each row corresponds to one NP.

Supplementary Data 2. Nanoparticles measured from HRTEM images. Long axis and short axis correspond to the maximum and minimum Feret diameters, respectively. The area was calculated in nm^2, and the Equivalent Circular Diameter (ECD) was derived from the area (see section SI-3).

Supplementary Data 3. Relative abundance of bacterial genera in the glycerol-amended microcosms B4 and B8. The water of the microcosms was sampled at the end of the 130-day incubation, when more than 90% of U(VI) was removed from the supernatant. Each microcosm was analysed with three replicates (n = 3). Average corresponds to the mean relative abundance calculated from six replicates (B4A–C and B8A–C), which exhibited similar geochemical conditions and U(VI) removal.
